# Two new species and a new combination in *Protium* (Burseraceae) from Costa Rica

**DOI:** 10.3897/phytokeys.76.10298

**Published:** 2017-01-18

**Authors:** Daniel Santamaría-Aguilar, Laura P. Lagomarsino

**Affiliations:** 1 Current address: Missouri Botanical Garden, P.O. Box 299, St. Louis, Missouri 63166-0299, USA; 2 Missouri Botanical Garden, P.O. Box 299, St. Louis, Missouri 63166-0299, USA, and University of Missouri–St. Louis, Biology Department, One University Blvd., Research Building, St. Louis, MO 63121, USA

**Keywords:** Burseraceae, Costa Rica, Nicaragua, Protium, Sapindales, taxonomy

## Abstract

Two new species of *Protium* (Burseraceae) are described and illustrated: *Protium aguilarii* **sp. nov.**, from the Pacific slope of the Osa Peninsula, Puntarenas Province, Costa Rica; and *Protium hammelii* **sp. nov.**, from wet forests on the Caribbean slopes of Nicaragua and Costa Rica. In addition, *Protium brenesii* **comb. nov.**, is proposed as a new combination based on *Trichilia brenesii*, a name that was based on a specimen collected with flowers in the mountains near San Ramón, Alajuela Province, Costa Rica. It is compared with *Protium costaricense*, a similar species with which it has been confused for more than 90 years. Finally, illustrations and specimen citations are provided for all the aforementioned taxa, and some others with which they have been confused.

## Abstract (es)

Se describen e ilustran dos nuevas especies de *Protium* (Burseraceae): *Protium aguilarii* **sp. nov.**, de la vertiente del Pacífico en la Península de Osa, provincia de Puntarenas, Costa Rica; y *Protium hammelii* **sp. nov.**, de los bosques húmedos de la vertiente del Caribe en Nicaragua y Costa Rica. Además, se propone la combinación *Protium brenesii* **comb. nov.**, basada en *Trichilia brenesii*, un nombre que fue descrito en base en un ejemplar con flores recolectado en las montañas de San Ramón, provincia de Alajuela, Costa Rica. Se compara con *Protium costaricense*, especie similar, con la cual se confundió por más de 90 años. Finalmente, se proveen ilustraciones y listas de los ejemplares examinados para todos los taxones antes mencionados, y además algunos otros similares.

## Introduction

The genus *Protium* (Burseraceae), with approximately 160 species, is nearly pantropical in distribution, though absent from continental Africa. Twelve species (including those described in this paper) have been recorded from Costa Rica, making this the largest of the five native genera in the country. *Protium* is distributed in Costa Rica from sea level to 1500 m, mainly in humid lowland forests, though some species occur in montane forest or (more rarely) relatively dry areas [e.g., Protium tenuifolium Engl. subsp. sessiliflorum (Rose) D. M. Porter on the Pacific slope]. The genus is characterized in general by its arborescent or less often shrubby habit; resin that is usually aromatic; imparipinnately compound, trifoliolate, or rarely unifoliolate leaves, with petiolules that are commonly pulvinulate at both ends, (3) 4–5-merous flowers with distinct or weakly connate petals and 8–10 stamens inserted outside the base of the nectary disk, and dehiscent fruits with 1–5 pyrenes.

The two new species described below and the need for a new combination in *Protium* were discovered during preparation of the Burseraceae treatment for the *Manual de Plantas de Costa Rica*. This study was based on an examination of *Protium* specimens deposited at A, CR, F, GH, MO and USJ (acronyms according Thiers 2016, continually updated), along with consultation of digital images in national and international virtual herbaria and relevant literature on *Protium* (e.g., Rose 1911; Standley 1937; Swart 1942; Standley and Steyermark 1946; Cuatrecasas 1957; Porter 1970; Daly 1987, 1989, 1997, 1999, 2002, 2014, 2016; Porter and Pool 2001). Additional field collections were conducted in Costa Rica in February through March 2016. The distribution map was generated using the program SimpleMappr (Shorthouse 2010) from coordinates reported on specimen labels; specimens whose label data did not indicate coordinates are shown in brackets.

## Taxonomy

### Protium aguilarii

Taxon classificationPlantaeSapindalesBurseraceae

D.Santam sp. nov.

urn:lsid:ipni.org:names:77159804-1

Figs 1 , 2 , 3

#### Diagnosis.

*Protium aguilarii* most closely resembles *Protium costaricense* (Rose) Engl. and *Protium pilosissimum* Engl., for their leaves with petiole, rachis, and leaflets that are hispidulous on the abaxial side and leaflets of comparable in size and coloration in herbarium material, but differs from both for its glabrous pistil and fruits (vs. pubescent).

**Figure 1.**
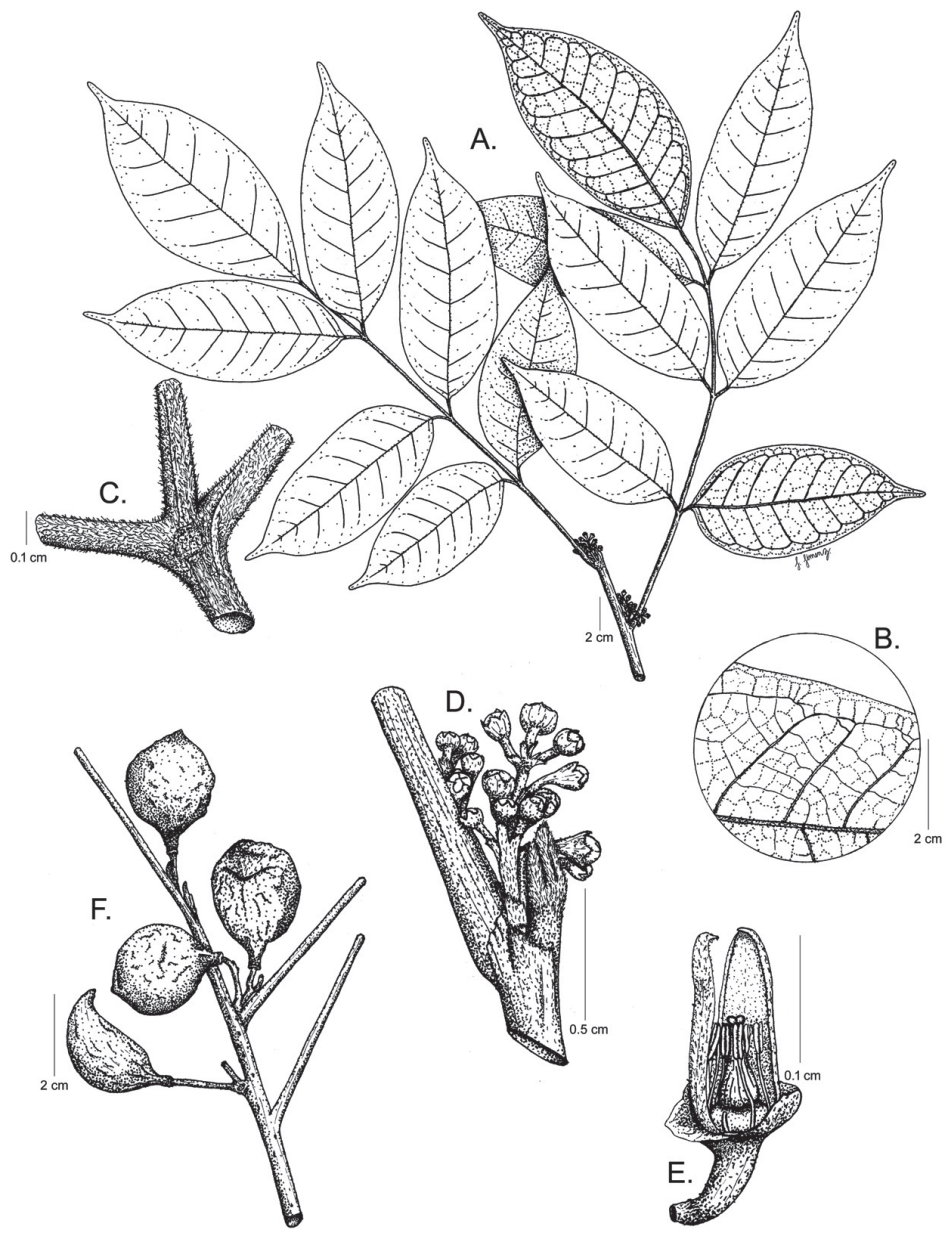
*Protium aguilarii*. **A** Branch with inflorescences **B** Venation **C** Pubescence on the leaf rachis and petiolules **D** Inflorescences **E** Female flower, with perianth partially removed **F** Fruits. **A–D** from *R. Aguilar 4593*, CR **E** from *R. Aguilar & X. Cornejo 11115*, CR **F** from *K. Thomsen 226*, CR. Drawing by Jessica Jiménez.

**Figure 2.**
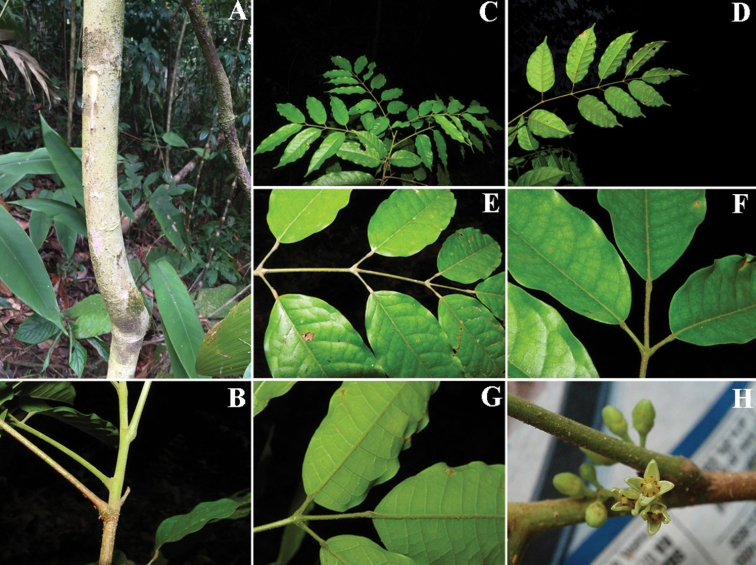
*Protium aguilarii*. **A** Trunk and bark **B** Twigs **C** Branch with leaves **D** Leaves showing abaxial side of leaflets **E** Leaf showing adaxial side **F** Leaflet bases **G** Venation **H** Flower. Photo credits: Reinaldo Aguilar (**A–G**) from *D. Santamaría et al. 9851*; and Xavier Cornejo (**H**) from *R. Aguilar & X.* Cornejo 11115.

**Figure 3.**
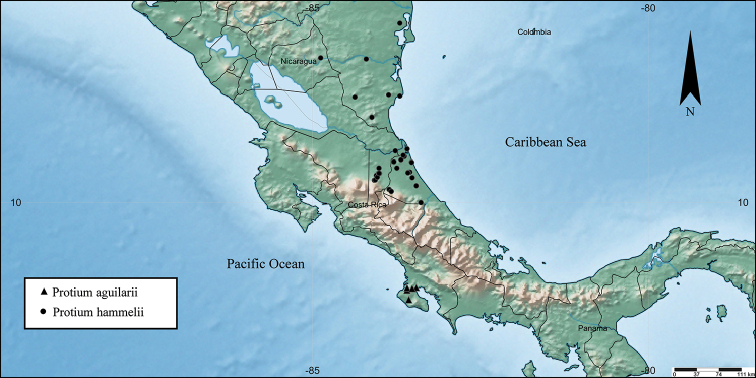
Distribution of *Protium aguilarii* and *Protium hammelii*.

#### Type.

COSTA RICA. Puntarenas: Reserva Forestal Golfo Dulce, Osa Peninsula, Rancho Quemado, ca. 15 km W of Rincón, on forested slopes at NW end of valley, near Fila Ganado, 08°33'N, 083°34'W, 300–400 m, 29 May 1988 (fr), *B. Hammel, G. Herrera, M. M. Chavarría & Á. Solís 16885* (holotype: MO-6664125!; isotypes: CR-51404! [ex-INB], NY-01189275, digital image!).

Tree, 8–15 m tall × 6–18 cm DBH, lacking stilt roots; external bark white (*D. Santamaría et al. 9851*). Resin transparent when fresh, a little sticky, aromatic. Twigs 2–4 mm diam, appressed-pubescent with simple or malpighiaceous, pale brown trichomes 0.05–0.6 mm long, sparsely lenticellate, solid, never stained white. Leaves 2–4-jugate, (12.5–) 19.5–33.8 cm long; petiole (2.3–) 3.3–6.3 cm long, 0.2 cm diam, semi-terete, smooth or slightly striate; rachis (2.6–) 3.5–11.8 cm long, terete, smooth or slightly striate; petiole and rachis hispidulous with simple, pale brown trichomes 0.1–0.5 mm long; lateral petiolules 0.3–1.7 cm long, with pulvinuli evident on both ends, smooth or slightly striate on both sides, rounded, hispidulous with yellowish brown trichomes; terminal petiolule 1.9–4.1 cm long, pulvinulus conspicuous; basal pair of leaflets 5.2–10 × 2–4.3 cm, elliptic to ovate, obtuse at the base; other lateral leaflets 6.7–13.2 × 2.6–5 cm, elliptic to ovate, obtuse to subcuneate at the base (with one side sometimes asymmetric); the terminal leaflet 7.3–14.2 × 2.7–5.8 cm, ovate, elliptic, base obtuse to subcuneate and symmetrical; apex acuminate, the acumen 0.7–1 cm long; margin entire; leaflets drying dark brown or olivaceous above and pale brown or olivaceous below; secondary venation brochidodromous, secondaries in 7–10 pairs of secondary veins, ascending, weakly arcuate, the spacing irregular, perpendicular intersecondaries sometimes present, intercostal tertiaries alternate or mixed percurrent tertiary; on abaxial side the midrib prominent, dense hispidulous, with trichomes 0.06–0.55 mm long, yellowish brown, the secondary veins prominent, with trichomes similar to the midrib, the higher-order veins prominulous, with scattered trichomes, the rest of the surface with scattered trichomes, not papillae; on adaxial side the midrib lightly prominent to flat, hispidulous, secondary veins flat, scattered hispidulous to almost glabrous, the higher-order veins flat almost glabrous, the rest of surface nearly glabrous. Inflorescences fasciculate, axillary (sometimes at leafless nodes), staminate inflorescences unknown, pistillate inflorescences ca. 0.6 cm long (0.9–1.2 cm in fruits), much shorter than the petiole, branching at the base, not flexuous, all axes densely pubescent with simple, yellowish brown or whitish trichomes; bracts subtending the inflorescences ca. 1.3 mm long, lanceolate, acuminate to obtuse at the apex, densely pubescent abaxially; those on primary axes ca. 0.6 mm long, broadly ovate, obtuse at the apex, densely pubescent abaxially; bracteoles subtending flowers ca. 0.8 mm long, triangular, acuminate at the apex, pubescent abaxially. Flowers 4-merous, the pedicel ca. 2.2 mm long (ca. 3 mm in fruit), sparsely pubescent with trichomes ca. 0.2 mm long. Staminate flowers unknown. Pistillate flowers with calyx 1–1.16 × 2.3 mm, sparsely pubescent on abaxial side with trichomes ca. 0.1 mm long, the lobes ca. 0.4 mm long, rounded to depressed-deltate, much taller than the disk, often persistent in fruit; petals white, ca. 2.6 × 0.9 mm, distinct, suberect at anthesis, lanceolate, sparsely appressed-pubescent on abaxial side with yellowish brown to whitish trichomes ca. 0.1 mm long, glabrous but papillose on the adaxial side, papillose and involute marginally, inflexed-apiculate at the apex (the apiculum ca. 0.2 mm long); disk ca. 0.4 tall × 0.2 mm thick, glabrous; staminodes 8, 1.66–1.8 mm long, the antepetalous almost equaling than antesepalous, the filaments ca. 0.9 mm long, flat, the anthers ca. 0.6 mm long, lanceolate, subcordate at the base; pistil ca. 1.5 × 1 mm (at the base), ovoid, glabrous, the style ca. 0.8 mm long, stigma lobes 4, globose, densely papillose. Fruits 1.6–1.8 × 1.2–1.4 cm, subglobose to slightly obliquely ovoid, green (possibly immature), obtuse at the base, acuminate at the apex, sometimes weakly curved, smooth and glabrous, stipitate (the stipe ca. 0.2 cm long); pseudoaril present, color unknown; pyrenes, 1 or 2 usually developing, 1.1–1.4 × ca. 1.2 cm, smooth, broadly ovate, obtuse at the base, acuminate at the apex, bony, the wall ca. 0.75 mm thick, yellowish; funicular scar ca. 0.7 cm long, usually not very deep, without a rib in the middle.

#### Habitat and distribution.

*Protium aguilarii* is endemic to Costa Rica, where it is restricted to the Osa Peninsula, on the southern Pacific coast in Puntarenas Province. It occurs in primary forest, at 150–400 m elevation. In Aguabuena, Rincón de Osa, this species occurs in well-drained forest on undulating terrain, with many palms and large lianas; here, it co-occurs with *Brosimum utile* (Kunth) Oken (Moraceae), *Carapa* Aubl. (Meliaceae), and *Symphonia* L. f. (Clusiaceae) (see, for example, *K. Thomsen 226*). In Rancho Quemado, *Protium aguilarii* is a small, infrequent tree on mountain ridges, where it is sympatric with tree species that are not very common in the area, or even the country as a whole, including *Faramea permagnifolia* Dwyer ex C. M. Taylor (Rubiaceae), *Hirtella papillata* Prance and *Licania corniculata* Prance (Chrysobalanaceae), *Oecopetalum greenmanii* Standl. & Steyerm. (Metteniusaceae), and *Ruptiliocarpon caracolito* Hammel & N. Zamora (Lepidobotryaceae).

#### Phenology.

*Protium aguilarii* is known from only six fertile collections (one of these with flowers in bud). Pistillate flowers have been collected in April, and fruits in February, May, June, and December.

#### Common name.

Copalillo (Spanish; Costa Rica, *K. Thomsen 683*).

#### Etymology.

The epithet of this new species honors Reinaldo Aguilar Fernández for his important contributions to botany and his dedicated study and devoted collection of the plants of the Osa Peninsula for more than 25 years. He has become the world’s expert in the flora of this remarkably species-rich and beautiful corner of the world. This species is further dedicated to him in appreciation of his support and intellectual stimulation.

#### Discussion.

*Protium aguilarii* can be recognized by the combination of leaves with 5–9 leaflets with hispidulous pubescence on the petiole, rachis, petiolules, abaxial side of the leaflets, and inflorescence axes; leaflets with a distinct marginal vein that is visible on the abaxial side; short inflorescences and infructescences; flowers that are 4-merous, with the petals appressed-pubescent on the abaxial side and glabrous on the adaxial side; and glabrous pistils. The new species resembles, and has been confused with, *Protium costaricense*, which, as treated here, is known only from the Caribbean slope of Nicaragua, Costa Rica, and Panama. Both species share hispidulous pubescence on the leaflets and inflorescences, but *Protium aguilarii* has a glabrous pistil (vs. densely pubescent in *Protium costaricense*), short inflorescence (ca. 0.6 vs. 1.6–6.5 cm long), petals that are glabrous on the abaxial side (vs. sparsely pubescent), and secondary venation brochidodromous (vs. eucamptodromous). *Protium aguilarii* also has usually shorter (non-basal) leaflets than *Protium costaricense* (6.7–13.2 vs. 10.5–17.5 cm), and glabrous fruits (vs. minutely pubescent with scattered trichomes), with a stipitate base, the stipe ca. 0.2 cm long (vs. sessile or with stipitate ca. 0.1 cm long). Additionally, *Protium aguilarii* differs from *Protium costaricense* by its smooth (vs. rugose) pyrene. In Costa Rica, others species with glabrous pistils or pistillodes are *Protium aracouchini* Marchand, *Protium hammelii* (described here), *Protium panamense* (Rose) I. M. Johnst., and *Protium ravenii* D. M. Porter. Unlike *Protium aguilarii*, these species have nearly glabrous leaves and other vegetative parts. *Protium aguilarii* also shares some similarities with the South American *Protium pilosissimum* including short inflorescences and pubescent leaflets but it differs by its pubescent pistil or pistillode and fruit (vs. glabrous in *Protium aguilarii*).

#### Additional material examined.

**Costa Rica. Puntarenas**: Osa, Bahía Chal, La Parcela, 08°43'50"N, 083°27'17"W, 150 m, 25 Jul 1996 (fl bud), *R. Aguilar 4593* (CR-2 sheets, F); Bahía Chal, La Parcela, 08°43'50"N, 083°27'17"W, 150 m, 12 Dec 1996 (fr), *R. Aguilar 4746* (CR); Rancho Quemado, camino a Drake, parte mas elevada del camino, 200 m al Este de la torre del ICE, en una trocha que lleva al Tierra de Conservación de Rancho Quemado, 08°41'33"N, 083°35'35"W, 350 m, 04 Apr 2008 (♀ fl), *R. Aguilar & X. Cornejo 11115* (MO, NY-digital image, USJ); Rancho Quemado, siguiendo a fila Ganado, 08°43'30"N, 083°35'30"W, 200–450 m, 26 Nov 1991 (st), *J. Marín & G. Herrera 306* (CR-2 sheets); Sierpe, Península de Osa, subiendo hacia el Cerro Chocuaco, desde el Bajo de San Juan, 400 m, 12 Jan 1991 (st), *C.O. Morales 262* (USJ); Distrito de Sierpe, Península de Osa, entre Rancho Quemado y Drake, trocha al sur, sobre la fila, antes de llegar a la torre, 08°41'30"N, 083°35'28"W, 375 m, 21 Mar 2016 (st), *D. Santamaría et al. 9851* (CR); Península de Osa, Aguabuena, 3.5 km W of Rincón, 1 km N of BOSCOSA station, 08°43'N, 083°31'W, 350 m, 09 Jan 1993 (fr), *K. Thomsen 226* (CR, NY-digital image, USJ); Península de Osa, Aguabuena, 3.5 km W of Rincón, 1 km N of BOSCOSA station, 08°43'N, 083°31'W, 350 m, 13 Nov 1992 (st), *K. Thomsen 683* (CR); Península de Osa, Aguabuena, 3.5 km W of Rincón, 1 km N of BOSCOSA station, 08°43'N, 083°31'W, 350 m, 30 May 1993 (st), *K. Thomsen 707* (CR); Península de Osa, Aguabuena, 3.5 km W of Rincón, 1 km N of BOSCOSA station, 08°43'N, 083°31'W, 350 m, 18 Jun 1993 (fr), *K. Thomsen 807* (CR, NY-digital image).

### Protium hammelii

Taxon classificationPlantaeSapindalesBurseraceae

D.Santam sp. nov.

urn:lsid:ipni.org:names:77159805-1

Figs 3 , 4 , 5 , 7A

#### Diagnosis.

*Protium hammelii* is similar to *Protium multiramiflorum* Lundell and *Protium panamense* for their nearly glabrous leaves, petals, and usually pistil or pistillode (always glabrous in *Protium panamense*; sometimes glabrous in *Protium multiramiflorum*). However, the new species it is distinguished from *Protium multiramiflorum* by the short calyx in both sexes (0.7–1.3 vs. 1.4–2 mm long), pyrenes with thick walls (0.6–1.1 vs. 0.3–0.5 [–0.6] mm thick), and a short and shallow scar (0.3–0.45 [–0.5] vs. 0.4–0.7 cm long). It is distinguished from *Protium panamense* by its smaller lateral (11–22.2 × 3.3–6.4 vs. 16–32.5 × 6.7–10.3 cm) and terminal (11.2–17.7 × 3.9–8.5 vs. 15–28.3 × 7–13.6 cm) leaflets and shorter petiolules.

#### Type.

COSTA RICA. Limón: Parque Nacional Tortuguero, 5 km N de La Aurora, Guápiles, límite sur del Parque, junto río Sierpe, 10°22'00"N, 083°31'00"W, 30 m, 11 Apr 1990 (♂ fl), *J. Solano 77* (holotype: CR-152860!; isotypes: CR-51496! [ex-INB], F-2 sheets 2081330!, 2127441!, MO-6664125!, NY-01189417, digital image!).

Tree, 4–30 tall × 9–40 cm DBH, sometimes with stilt roots; external bark grayish. Resin transparent when fresh, aromatic. Twigs 2–5 mm diam, appressed-pubescent with simple or malpighiaceous, whitish yellow trichomes 0.1–0.5 mm long, to glabrescent, sparsely lenticellate, solid, never white-stained with resin that crystallizes on the stem (except weakly in *W.D. Stevens 31653*; also on the fruits). Leaves (2–) 4–6 jugate, 21.5–43.5 cm long; petiole (2.7–) 4.7–7.4 (–8.2) cm long, 0.1–0.3 cm diam, semi-terete except weakly sulcate at the base, striate; rachis 4–8.2 (–9) cm long or absent, terete, striate on both sides; petiole and rachis nearly glabrous or sparsely pubescent with simple and malpighiaceous, usually whitish yellow trichomes 0.1–0.3 mm long; lateral petiolules 0.7–2.1 cm long, with pulvinuli evident on both ends, striate, canaliculate adaxially, glabrous or minutely pubescent with whitish yellow trichomes; terminal petiolule 1.8–4.3 (–5.5) cm long, pulvinulus conspicuous; basal pair of leaflets 10.1–19 × 3.7–6.7 cm, ovate or lanceolate, obtuse to subcuneate at the base (sometimes asymmetric); other lateral leaflets 11–22.2 × 3.3–6.4 cm, lanceolate, oblong, ovate or elliptic, obtuse to subcuneate at the base; the terminal 11.2–17.7 × 3.9–8.5 cm, broadly elliptic to obovate, lanceolate, obtuse or subcuneate at the base (usually with both sides equal); apex acuminate, the acumen 0.7–1.3 cm long; margin entire or much more commonly sparsely denticulate; leaflets drying more or less pale brown, olivaceous to amber on both sides; secondary venation eucamptodromous or slightly brochidodromous, secondaries in 12–17 pairs of secondary veins, ascending, weakly arcuate, sometimes discolored on abaxial side, ascending, the spacing irregular, perpendicular intersecondary veins often 1 per pair of successive secondary veins or absent, intercostal tertiaries alternate or mixed percurrent tertiary; on abaxial side the midrib prominent, glabrous or minutely pubescent, with trichomes ca. 0.03–0.6 mm long, whitish yellow or reddish, secondary veins prominent, with trichomes similar to the midrib or glabrous, the higher-order veins prominulous with scattered trichomes or glabrous, the rest of surface almost glabrous to glabrous, not papillate; on adaxial side, the midrib prominent, minutely and scattered pubescent, secondary veins impressed to flat with trichomes similar to the midrib or glabrous, the higher-order veins flat to lightly impressed, with scattered trichomes, the rest of surface with scattered trichomes to glabrous. Inflorescences axillary (sometimes at leafless nodes), generally branching at the base, not flexuous, the staminate inflorescences 3.4–8.5 cm long, shorter or exceeding the petiole, minutely pubescent with simple and malpighiaceous, whitish trichomes on all axes, the pistillate inflorescences 1–1.8 cm long [(1.4)– 2.5–12.5 cm in fruit], much shorter than the petiole; bracts subtending the inflorescences 1–1.7 mm long, triangular, acuminate at the apex, densely pubescent abaxially; those on primary axes 0.6–1.3 mm long, triangular, acuminate or obtuse at the apex, densely pubescent abaxially; bracteoles subtending flowers 0.3–0.8 mm long, triangular or broadly ovate, obtuse to acuminate at the apex, pubescent or nearly glabrous abaxially. Flowers 4(5)-merous, the pedicel 1.5–2.8 mm long (2–6 mm in fruit), generally glabrous. Staminate flowers with calyx 0.7–1.3 × 1.3–2.4 mm, much taller than the disk, the lobes 0.4–1 mm long, rounded to depressed-deltoid, glabrous on abaxial side, papillate marginally, often persistent in fruit; petals variously reported as green, greenish yellow or white, 3–4 × 1.4–1.8 mm, distinct, erect to suberect at anthesis, lanceolate or ± triangular, glabrous on the abaxial side, glabrous but papillose on the adaxial side, papillose and weakly involute marginally, inflexed-apiculate at the apex (the apiculum 0.15–0.25 mm long); disk 0.23–0.46 tall × 0.26–0.5 mm thick, glabrous; stamens 8, (sub) equal, the antesepalous 1.8–2.4 mm long, the antepetalous 1.5–2 mm long, exceeding the pistillode, the filaments more or less flat, papillate, the anthers 0.7–0.9 mm long, lanceolate, obtuse to subcordate at the base, apiculate at the apex; pistillode 0.5–0.83 × 0.5–0.8 mm at the base, ovoid, globose or conical, glabrous, the style 0–1.5 mm long, stigma lobes 4, subglobose to weakly angulate, densely papillose. Pistillate flowers with calyx 1–1.2 × 2–2.5 mm, the lobes ca. 0.8 mm long, all parts similar to that of the staminate flowers; petals green, greenish yellow or white, distinct, 3–3.5 × 1.16–1.5 mm, suberect to reflexed at anthesis similar to those of the staminate flowers; disk 0.4–0.5 tall × ca. 0.3 mm thick, glabrous; staminodes 8, (sub) equal, the antesepalous 1.5–1.7 mm long, the antepetalous 1.3–1.5 mm long, shorter or longer than the pistil, the filaments flat, not papillate, the anthers 0.6–0.8 mm long, lanceolate, cordate at the base; pistil 1.16–1.3 × 1–1.23 mm (at the base), ovoid or conical, glabrous, the style 0.3–0.8 mm long, stigma lobes-4, globose, densely papillose, each lobe sulcate on the middle (± as an inverted “C”). Fruits 1.6–2.4 × 1.1–2.1 cm, globose to ovoid, reddish or green (*M. Ballestero 71*) when ripe, obtuse at the base, the apex generally conspicuously apiculate at the apex, smooth or (more commonly) lenticellate, glabrous, stipitate [the stipe (0.1–) 0.2–0.5 cm long]; pseudoaril white; pyrene 1(2), 1.3–1.6 × 0.9–1.2 cm, smooth, ovoid to very widely ovate, obtuse at the base, apiculate at the apex, the wall 0.6–1.1 mm thick, whitish or yellowish; funicular scar 0.3–0.45 (–0.5) cm long, usually not very deep, without a rib in the middle or the rib inconspicuous.

#### Habitat and distribution.

This species is known so far only from wet forest on the Caribbean slope of Nicaragua and Costa Rica. It occurs mainly between 10 and 200 m in elevation, although some collections were made between 300 and 700 m. In Costa Rica, this species is common in the Sarapiquí region, where it seems to prefer alluvial soils on flat or relatively flat ground, sometimes on river banks. In this area, *Protium hammelii* grows sympatrically with the following species: *Carapa guianensis* Aubl. (Meliaceae), *Dipteryx panamensis* (Pittier) Record & Mell (Fabaceae), *Euterpe precatoria* Mart. (Arecaceae), *Minquartia guianensis* Aubl. (Coulaceae), and *Pentaclethra macroloba* Kuntze (Fabaceae), among others species (N. Zamora, pers. comm.; May 2016).

#### Phenology.

In Nicaragua, *Protium hammelii* has been collected with fruits in January, February, from May to July, and in October, but never in flower. In Costa Rica, it has been collected with staminate flowers in January, February, April, and December; pistillate flowers in January and February; and fruits in January, March, April, June, from August to October, and in December.

#### Common name.

Alcanfor (Spanish; Nicaragua, *R. Rueda et al. 2642, 2701*; *J.C. Sandino 3424*).

#### Etymology.

The specific epithet honors Barry E. Hammel, curator at the Missouri Botanical Garden and co-editor of the *Manual de Plantas de Costa Rica*, in recognition of his extensive work on the Costa Rican flora, as well as his personal support and motivation.

#### Discussion.

*Protium hammelii* is recognizable by its almost glabrous vegetative parts, leaves with 5–7 leaflets, commonly with a sparsely denticulate margin, prominent tertiary veins on the abaxial side, 4(5)-merous flowers with glabrous petals, pistils, and pistillodes, the pistillate flowers with globose stigma lobes that are sulcate in the middle, and glabrous, usually lenticellate fruits that are stipitate and apiculate at the apex. Specimens of *Protium hammelii* have frequently been identified as *Protium glabrum* (Rose) Engl., a species that is widespread from Belize to Panama, or *Protium panamense*, from Costa Rica, Panama, and Colombia. The first of these is common in Costa Rica, while the second is quite rare (Fig. 6A–F); both have leaflets that are always entire. *Protium hammelii* differs from *Protium glabrum* by its consistently glabrous petals on both sides, pistil, and pistillode (vs. petals on the adaxial side, pistil, and pistillode always pubescent). Fruiting material can usually be distinguished by the apiculate apex of the fruits of *Protium hammelii* (vs. obtuse or rounded), and the glabrous (vs. with very small trichomes). *Protium panamense* shares occasional stilt-roots, glabrous flowers and fruits that are apiculate at the apex with *Protium hammelii*, but *Protium hammelii* usually has smaller lateral (11–22.2 × 3.3–6.4 vs. 16–32.5 × 6.7–10.3 cm), and terminal leaflets (11.2–17.7 × 3.9–8.5 vs. 15–28.3 × 7–13.6 cm) that are also usually thinner, as well as thinner lateral and terminal petiolules that are also shorter (1.8–4.3 [–5.5] vs. 5.2–8.7 cm); *Protium hammelii* further has smaller (1.6–2.4 × 1.1–2.1 cm), globose to ovoid fruits (vs. usually 2.2–3 × 0.9–1.7 cm lanceolate. *Protium multiramiflorum* from Mexico to Honduras is similar to *Protium hammelii* in its nearly glabrous leaves, petals, and sometimes pistil or pistillode. (Although the pistil of *Protium multiramiflorum* was originally described as glabrous [Lundell 1937], almost all collections studied, including one of the isotypes [*Lundell 6212*, GH-2 sheets!] and the paratype [*Schipp 1021*; A!, F!, GH!, MO!], have the pistil or pistillode with tiny, scattered trichomes). The new species differs in its short calyx in both sexes (0.7–1.3 vs. 1.4–2 mm long), stigma lobes that are sulcate (vs. not sulcate), and pyrenes with thick walls (0.6–1.1 vs. 0.3–0.5 [–0.6] mm thick) and short scar (0.3–0.45 [–0.5] vs. 0.4–0.7 cm long) (Fig. 7B). Others species in Costa Rica with glabrous pistils or pistillodes are *Protium aracouchini* (Figs 6G–J), *Protium ravenii* (Figs 6K–N), and *Protium aguilarii*. The first two species can be distinguished from *Protium hammelii* by their flexuous inflorescences and twigs and abundant exuding resin that becomes whitish and chalky (Figs 6H and L, inset), while *Protium aguilarii* can be distinguished by its petals that are pubescent abaxially.

**Figure 4.**
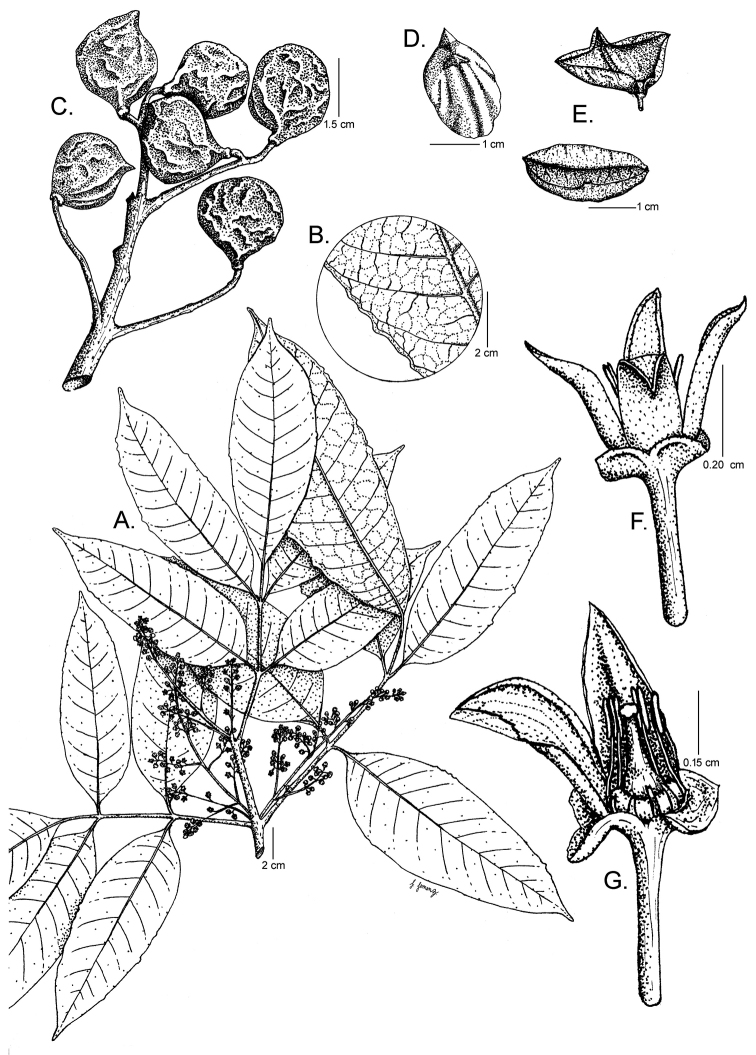
*Protium hammelii*. **A** Branch with inflorescences **B** Venation on the abaxial side and marginal teeth of leaflets **C** Fruits **D** Pyrene **E** Fruit valves **F** Flower **G** Staminate flower with two petals removed, showing the pistillode and stamens. **A** and **B** from *J. Solano 77*, CR **C–E** from *J. Gómez-Laurito et al. 10998*, CR **F, G** from *W.D. Stevens 23769*, CR. Drawing by Jessica Jiménez.

**Figure 5.**
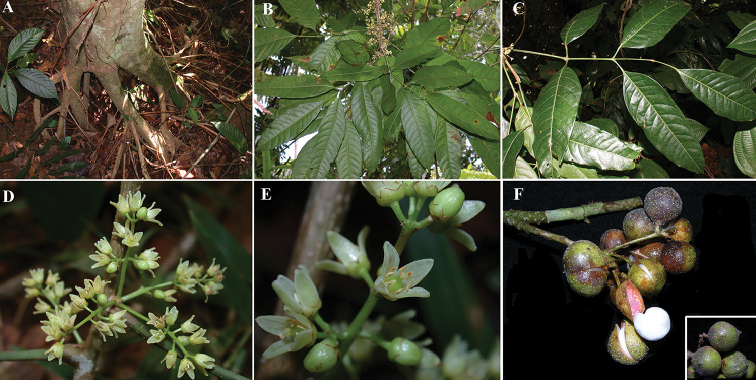
*Protium hammelii*. **A** Stilt roots **B** Branch with inflorescences **C** Adaxial side of the leaflets **D** Inflorescences **E** Flower **F** Fruits. Photo credits: Orlando Vargas (**A–E**); and N. Zamora (**F**).

**Figure 6.**
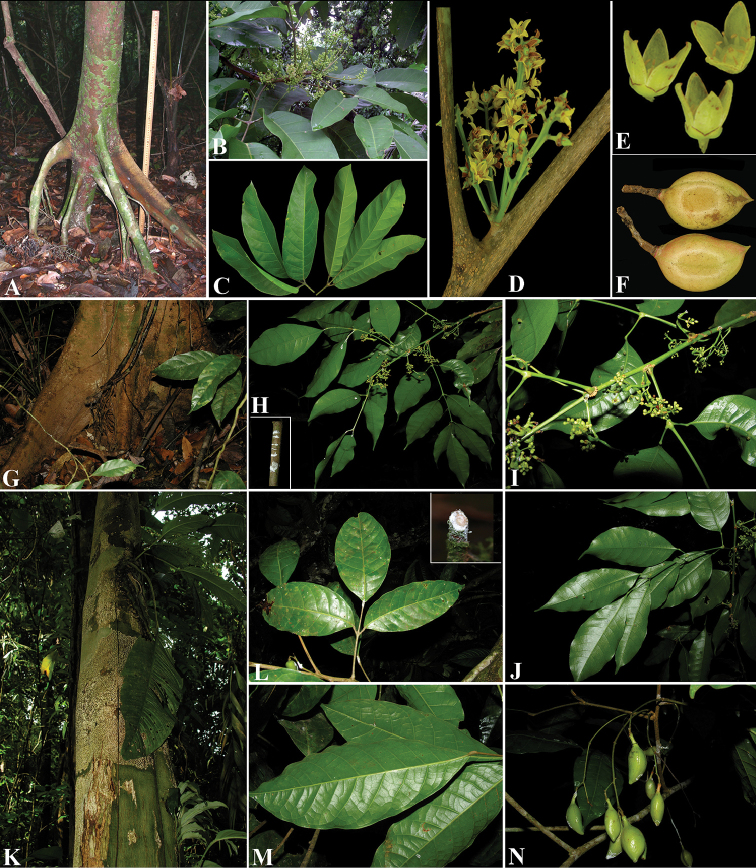
*Protium panamense*. **A** Stilt roots **B** Branch with inflorescences **C** Leaflets **D** Inflorescences **E** Staminate flowers **F** Fruits. *Protium aracouchini* **G** Trunk base **H** Branch with inflorescences; inset showing dry resin on cut twig **I** Inflorescences; also see the suberose petiole base **J** Abaxial side of the leaflets. *Protium ravenii* **K** Trunk **L** Adaxial side of the leaflets; inset dry resin on cut twig **M** Abaxial side of the leaflets **N** Fruits. Photo credits: Rolando Pérez (**A**); Carmen Galdames (**B**); Steven Paton (**C–F**). **G–J** photos by Reinaldo Aguilar, from *D. Santamaría & R. Aguilar 9836* **K–N** photos by Reinaldo Aguilar, from *R. Aguilar 12067*, except **L**, inset by Orlando Vargas.

**Figure 7.**
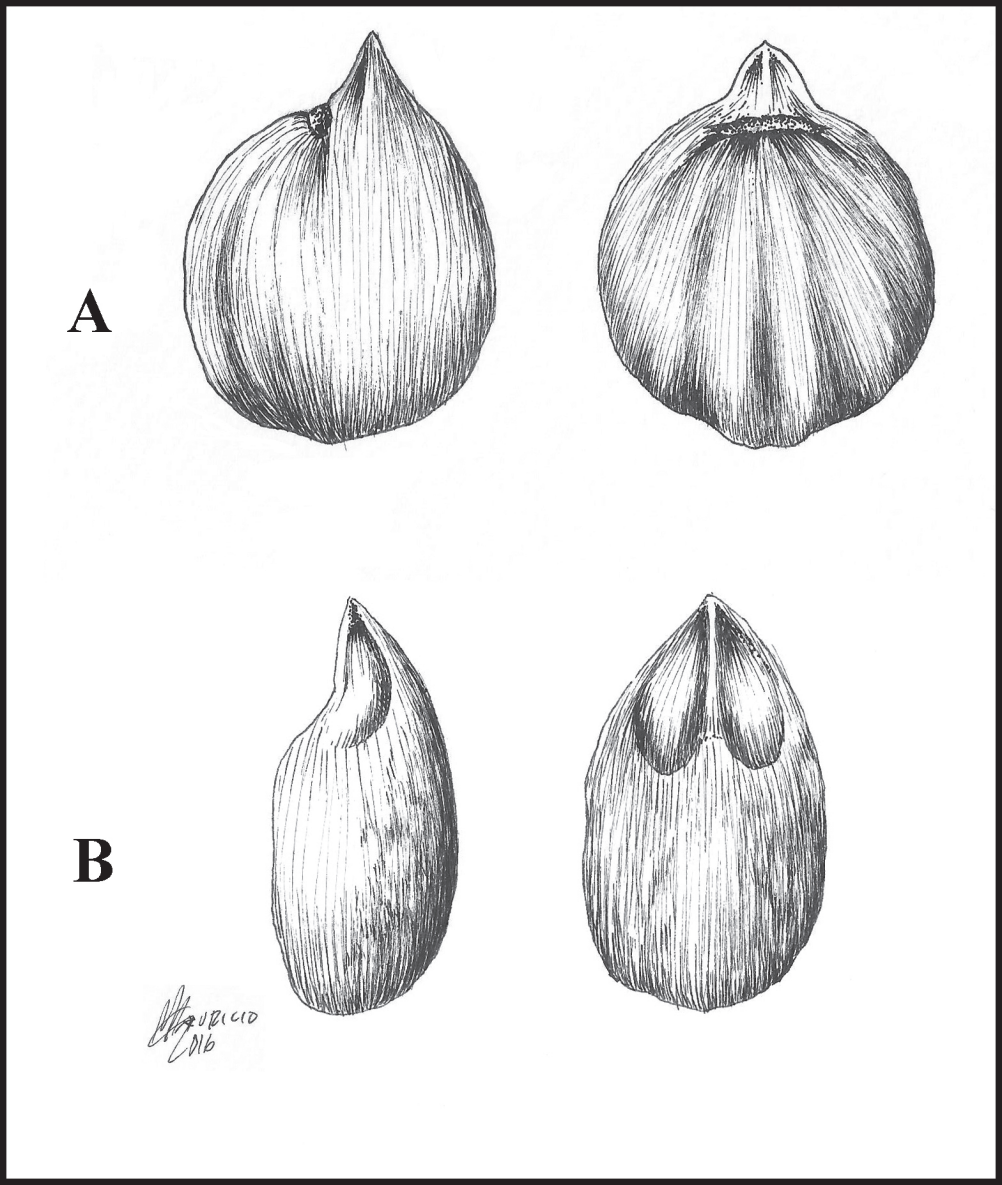
Comparison between the pyrenes of *Protium hammelii* (**A**) and *Protium multiramiflorum* (**B**). **A** from *G. Davidse & G. Herrera 30879*; and **B** from *G.M. Aguilar et al. 4754*. Drawing by Alex M. Campos.

Fruit dispersal by birds and mammals has been reported at the La Selva Biological Station for *Protium panamense* (Vargas 2000), but the observation likely corresponds to *Protium hammelii*.

#### Additional material examined.

**NICARAGUA. Atlántico Norte** [Zelaya]: Reserva Bosawas, Mpio. de Bonanza, Cerro Cola Blanca, entre el cacerío de Vitinia y empalme de la Comarca de Panamá, 14°04'N, 084°34'W, 200 m, 02 Jun 1997 (fr), *R. Rueda & I. Coronado 6595* (MO). **Atlántico Sur** [Zelaya]: área de la Bahía de Bluefields, río Escondido, camino entre El Pool y Abardeen Hills, 0–30 m, 22 Mar 1949 (st), *A. Molina 1899* (F); Monkey Point, Caño El Pato, 1.5 km sobre la ribera del Caño, 11°35'N, 083°42'W, 10 m, 25 Oct 1981 (fr), *P.P. Moreno 12411* (MO); Caño Montecristo, al este del Campamento Germán Pomares, 11°36'N, 083°52'W, 60–90 m, 08 Feb 1982 (fl bud), *P.P. Moreno 15132* (MO); Mpio. de Rama, Loma Buena Vista, 12°08'N, 084°12'W, 100–150 m, 23 May 1984 (st), *W. Robleto 613* (MO); a lo largo del río Maíz, 11°16'N, 084°07'W, [25–50 m], 08 Jan 1995 (fr), *R. Rueda et al. 2642* (MO); Mpio. Laguna de Perla, río Wawanshang, 12°40'N, 083°42'W, 50 m, 15 Feb 2002 (fr), *R. Rueda & R. Dolmus 16851* (MO); 1 km de Colonia Serrano, río Serrano, 11°34'N, 084°22'W, 70–80 m, 31 Jul 1982 (fr), *J.C. Sandino 3424* (MO). **Chontales**: Along road from Ciudad Sandino toward El Guabo, 0.7 km SW of El Porvenir, 12°09'02"N, 084°52'43"W, 345 m, 17 May 2011 (fr), *W.D. Stevens 31653* (MO). **Matagalpa**: Falda norte del Cerro Musún, frente a trocha a Wanawás, [13°02'N, 085°15'W], 200–500 m, 16 May 1980 (fr), *M. Araquistain & P.P. Moreno 2789* (MO). **Río San Juan**: Mpio. de San Juan del Norte, del Delta 1 km al este y después 2 km al norte, 10°46'N, 083°46'W, [40–80 m], 08 Jun 1995 (st), *R. Rueda et al. 2701* (MO). **Costa Rica. Alajuela**: Los Chiles, Finca La Urraca, Los Lirios, camino a los Chiles, ca. 100 m, 11 Dec 1985 (fr), *J. Gómez-Laurito et al. 10998* (CR, F, USJ). **Heredia**: Sarapiquí, OET, La Selva, 14 Jun 2004 (fr), *R. Aguilar et al. 8337* (LSCR-digital image); Parque Nacional Braulio Carrillo, frente al Puesto La Ceiba, 10°19'47"N, 084°04'48"W, 400–700 m, 23 Dec 1988 (fr), *M. Ballestero 71* (CR, MO); about 5 km north of Puerto Viejo along the road to El Muelle, 10°28'N, 083°58"W [10°30'36"N, 084°00'36"W], 100 m, 08 Jan 1967 (♂ fl), *W.C. Burger & G. Mata 4307* (F-2 sheets, MO); about 5 km north of Puerto Viejo along the road to El Muelle, 10°28'N, 083°58"W [10°30'36"N, 084°00'36"W], 100 m, 08 Jan 1967 (♀ fl, fr), *W.C. Burger & G. Mata 4315* (F, MO, NY-digital image); Finca La Selva, the OTS Field Station on the río Puerto Viejo just E of its junction with the río Sarapiquí, Southwest trail, 1600 m line, 100 m, 16 Feb 1981 (♂ fl), *J.P. Folsom 8965* (F, MO); Finca La Selva, the OTS Field Station on the río Puerto Viejo just E of its junction with the río Sarapiquí, central trail to Holdridge Trail 3000 m line, 100 m, 08 Mar 1981 (immat fr), *J.P. Folsom 9279* (MO); Finca La Selva, the OTS Field Station on the río Puerto Viejo just E of its junction with the Río Sarapiquí, junction South Boundary and Western Boundary, 21 Mar 1981 (immat fr), *J.P. Folsom 9435* (CR); Finca La Selva, the OTS Field Station on the río Puerto Viejo just E of its junction with the Río Sarapiquí, El Swampo Trail, 100 m, 27 Apr 1981 (fr), *J.P. Folsom 9882* (F, MO, NY-digital image); Magsasay, near La Selva, 10°24'N, 084°03'W, 150 m, 16 Jul 1990 (st), *A.H. Gentry 71766A* (MO); La Selva, río Sarapiquí near Puerto Viejo, junction SSO and LOC Trails, 10°26'N, 084°01'W, 100 m, 05 Jan 1993 (all st), *A.H. Gentry et al. 78485* (CR, MO), *78507* (CR, MO), *78522* (CR, MO), *78543* (CR, MO); La Selva, río Sarapiquí near Puerto Viejo, junction SSO and LOC Trails, 10°26'N, 084°01'W, 08 Jan 1993 (st), *A.H. Gentry & R. Ortiz 78630* (CR, MO); Parque Nacional Braulio Carrillo, fila Carrillo, 700 m, 30 Mar 1984 (fr), *L.D. Gómez et al. 21135* (CR); Parque Nacional Braulio Carrillo, estación El Ceibo, 10°20'00"N, 084°04'00"W, 450–500 m, 13 Mar 2003 (fr), *J. González 3147* (CR); Parque Nacional Braulio Carrillo, estación El Ceibo, 10°20'00"N, 084°04'00"W, 450–500 m, 14 Mar 2003 (immat fr), *J. González 3176* (CR, MO, USJ); Chilamate, Cerros de Sardinal, finca propiedad de Isaias Alvarado, 100 m, 26 Aug 2007, *J. González et al. 9326* (LSCR-digital image); Finca La Selva, Arboretum tag #522, 10°26'N, 084°01'W [10°25'53"N, 084°00'13"W], [40 m], 30 Dec 1970 (♂ fl bud), *G.S. Hartshorn 968* (F, MO-2 sheets); Finca La Selva, 10°26'N, 084°01'W, [100 m], 25 Jan 1973 (fr), *G.S. Hartshorn 1108* (CR); Finca La Selva, Holdridge Arboretum tag #119, 10°26'N, 084°01'W, [100 m], 22 Aug 1975 (fr), *G.S. Hartshorn 1476* (CR, F, LSCR-digital image, MO); Finca La Selva, the OTS Field Station on the río Puerto Viejo just E of its junction with the río Sarapiquí, quebrada El Sura, 100 m, 06 Jun 1984 (fr), *B. Jacobs 2148* (CR); Finca La Selva, the OTS Field Station on the río Puerto Viejo just E of its junction with the río Sarapiquí, camino circular Lagano, at bridge across Q. [Quebrada] Salto, 100 m, 15 Jun 1928 (fr), *B. Jacobs 2362* (F); Finca La Selva, the OTS Field Station on the río Puerto Viejo just E of its junction with the río Sarapiquí, vicinity Sendero Jagarunda and Lindero Sur intersection, [10°25'53"N, 084°00'13"W], 100 m, 28 Jun 1984 (fr), *B. Jacobs 2644* (MO); río Sarapiquí, 125 m, 18 Jan 1966 (fl bud), *A. Jiménez 3602* (F-2 sheets, CR); Finca Hermanos Vargas, 1 km al Suroeste de Puerto Viejo, 125 m, 04 Feb 1966 (♂ fl), *A. Jiménez 3602* (CR); Estación Biológica La Selva, LOC 600, 10°25'47"N, 084°01'00"W, 55 m, 29 Jul 2004 (st), *S. Letcher 77* (USJ); Finca La Selva, the OTS Field Station on the río Puerto Viejo just E of its junction with the río Sarapiquí, Far Loop Trail at about 350 m, [10°25'53"N, 084°00'13"W], 100 m, 11 Feb 1996 (♂ fl bud), *R.L. Wilbur 65080* (F, MO); Parque Nacional Braulio Carrillo, El Ceibo, 10°22'29"N, 084°02'10"W, 200–300 m, 24 Aug 2004 (fr), *R. Kriebel et al. 4857* (CR); Estación Biológica La Selva, 10°26'00"N, 084°00'30"W, 0–100 m, 05 Feb 2004 (st), *A. Rodríguez 8395* (CR, USJ); OET La Selva, a orillas del río Sarapiquí, [10°25'53"N, 084°00'13"W], [100 m], 30 Oct 2005 (fr), *N. Zamora 3871* (LSCR-digital image, MO). **Limón**: Bosque Lluvioso [finca propiedad de INBio], 10°11'28"N, 083°51'28"W, 350 m, 16 Aug 2005 (st), *L. Acosta et al. 3550* (CR); Pococí, 300 m al sur del Hotel Vista Al Mar, Tortuguero, 10°35'51"N, 083°31'40"W, 10 m, 22 Oct 2011 (fr), *M. Argueta 107, 109* (USJ); Guápiles, La Leona, 10°09'45"N, 083°49'37"W, 478 m, 31 May 2005 (fr), *C. Benavides & A. Chacón 160* (USJ); North shore of the mouth of the río Colorado at Barra del Colorado, 10°47'40"N, 083°35'30"W, 1–5 m, 12 Sep 1986 (fr), *G. Davidse & G. Herrera 30879* (CR, F, MO); Guápiles, Cariari, El Zota, Finca El Progreso, 10°30'35"N, 083°44'39"W, 40 m, 11 Jun 2011 (fr), *M. Díaz s.n.* (USJ-99607); Parque Nacional Tortuguero, Cerro Tortuguero, 10°35'37"N, 083°31'31"W, 5–120 m, 22 Oct 2011 (fr), *J. Gómez-Laurito 15689* (USJ); near río Parismina, 8 km W of Dos Bocas, [10°14'46"N, 083°27'21"W], 8 m, 31 Mar 1972 (fr), *R.W. Lent 2457* (CR, F, MO); Matina, Colonia Puriscaleña, Sendero Cerro Azul, 09°59'44"N, 083°23'08"W, 400–500 m, 15 Mar 2000 (fr), *E. Mora 984* (CR); Parque Nacional Tortuguero, Estación Agua Fría, rumbo Noreste, a orillas del río Agua Fría, 10°27'N, 083°33'W, 40 m, 02 Feb 1988, *R. Robles 1586* (CR, MO, NY-digital image); Llanura de Santa Clara, Chiporrisito, 10°36'10"N, 083°47'20"W, 400 m, 30 Jan 1995 (fl bud), *A. Rodríguez 507* (INB); Parque Nacional Tortuguero, Estación Agua Fría, Sendero El Aguacate, a 500 m de la entrada, 10°26'40"N, 083°34'40"W, 20 m, 11 Jan 1990 (fr), *J. Solano 62* (CR, MO, NY-digital image); Caño Chiquero, Tortuguero, 31 Jan 1986 (♂ fl), *R. Soto 2758* (CR); Cerro Coronel, E of Laguna Danto, 10°41'N, 083°38'W, 20–170 m, 16–13 Jan 1986 (♂ fl), *W.D. Stevens 23769, 23770* (CR, MO); Cerro Coronel, along río Colorado at and below outflow of Laguna Danto, 10°42'N, 83°39'W, 5–10 m, 25 Jan 2016 (fr), *W.D. Stevens 24002* (CR, MO); Cerro Coronel, E of Laguna Danto, 10°41'N, 083°38'W, 16 Mar 1987 (st), *W.D. Stevens et al. 24912* (MO); Monte Verde, 300 [ft?], 25 Apr 1928 (fr), *H.E Stork 1682* (F); Zapota Dos, ca. 20 NW of Tortuguero village, on farm of Ronulfo Vargas, 10°38'N, 083°41'W, 90–110 m, 16 Mar 1995 (st), *K. Thomsen 1377* (CR); Parque Nacional Tortuguero, Agua Fría, 10°26'35"N, 083°34'38"W, 32 m, 14 Jun 2007 (fr), *L.D. Vargas et al. 2379* (CR); Parque Nacional Tortuguero, Agua Fría, 10°26'20"N, 083°34'47"W, 30 m, 18 Oct 2007 (fr), *L.D. Vargas et al. 2813* (CR).

### Protium brenesii

Taxon classificationPlantaeSapindalesBurseraceae

(Standl.) D.Santam comb. nov.

urn:lsid:ipni.org:names:77159806-1

Figs 8A , 9

Basionym: Trichilia brenesii Standl. Publ. Field Mus. Nat. Hist., Bot. Ser. 18: 583. 1937. Type. COSTA RICA. [Alajuela:] colinas del Tremendal (San Pedro) de San Ramón, [09] Apr 1935 [♂ fl], *A.M. Brenes 20510* (holotype: F-866066!; isotypes: CR-2 sheets! [Hb. Brenes 20009, both with the same herbarium number], NY-00054791, digital image!).

#### Habitat and distribution.

*Protium brenesii* is only known from Costa Rica, where it grows mainly in the Cordilleras de Guanacaste, de Tilarán and Central on both the Caribbean and Pacific slopes, though it also has been collected in the Cordillera de Talamanca (Dota region) and the Valle del General. It is found in primary forest and along roads and rivers between 640 and 1500 m elevation. *Protium brenesii* is found at the highest elevations of any species of its genus in Costa Rica.

#### Phenology.

Collections with male flowers have been made from March to May, and December; female flowers in February; and fruits in March and April, and from June to December.

#### Common name.

Copal (Spanish; Costa Rica, *E. Bello 473*).

#### Discussion.

In the course of examining material identified as *Protium costaricense*, a notable number of collections from Costa Rica, mainly from 640–1500 m elevation in the Cordilleras de Guanacaste, de Tilarán and Central, were identified that differed from the rest. This material has twigs and leaflets with inconspicuous pubescence on the abaxial side; leaves with more numerous and usually narrower leaflets; and longer inflorescences and infructescences. *Protium costaricense*, as interpreted here, is a species of the lowlands of the Caribbean slope in Nicaragua, Costa Rica, and Panama, while the other collections represent a distinct montane taxon. An appropriate name already exists, and had been applied to some material collected in the Costa Rican cordilleras: *Trichilia brenesii* Standl. (1937: 583). Therefore, a new combination is proposed here, transferring *Trichilia brenesii* to *Protium*.

The first known collection of *Protium brenesii* was made by Alberto M. Brenes (1870–1948) in the mountains near San Ramón, Alajuela Province, Costa Rica, in May 1923 (*Brenes 19953*). This species is similar in some aspects to *Protium costaricense*, with which it has been confused for more than 90 years. These two taxa share the following morphological characteristics: twigs and leaves with dense pubescence; entire leaflets; 4(5)-merous flowers with pubescent petals, pistil, and pistillode; and rugose pyrene. The leaflets and fruits of the two species are also more or less similar in shape and size, but tend to be narrower in *Protium brenesii*. However, *Protium brenesii* can be distinguished from *Protium costaricense* by its longer inflorescences [(5–) 7–11.5 vs. 1.6–6.5 cm], with malpighiaceous and simple trichomes (vs. only simple) on the axes, and male and female flowers with the disk equal or taller than the calyx (vs. shorter), a feature that is also evident on collections with fruits. Additionally, the terminal leaflets of *Protium brenesii* are smaller (6.8–10.7 × 2.5–3.7 vs. 10.5–17.5 × 4.5–7.7 cm) and have shorter petiolules (1.5–2.5 vs. 2.8–3.5 [–5] cm). Importantly, *Protium brenesii* is a species of montane forests at elevations of 640–1500 m, while *Protium costaricense* is most frequent in lowland wet forests from 0–200 m. Some collections of *Protium brenesii* have been confused with *Protium confusum* (Rose) Pittier (or its synonym, *Protium schippii* Lundell), the latter distinguished by its distally undulate or serrulate leaflets (vs. entire), inflorescences usually with a mixture of malpighiaceous, reddish trichomes and apparently glandular, whitish trichomes (vs. yellowish to pale brown malpighiaceous and simple trichomes), and flowers with the petals, pistil, and pistillode densely covered by dark red trichomes (vs. with whitish or pale brown trichomes).

In the checklist of Plantas Vasculares de Monteverde (Haber 2014), *Protium* sp. A. (7508 [*W. Haber & E. Cruz*]) and *Protium costaricense* (*E. Bello 473*) correspond to *Protium brenesii*; the same applies to the collection cited by Gómez-Laurito and Ortiz (2004) (*J. Gómez-Laurito et al. 12278*).

**Figure 8.**
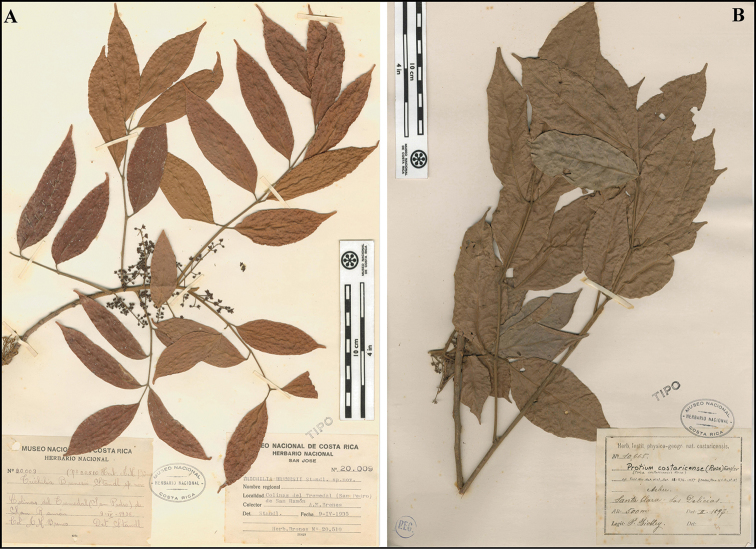
Types of *Protium brenesii* (**A**) and *Protium costaricense* (**B**). Images courtesy of Museo Nacional de Costa Rica.

**Figure 9.**
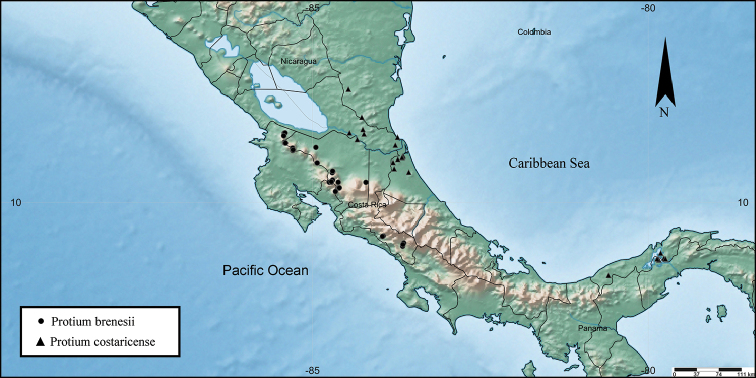
Distribution of *Protium brenesii* and *Protium costaricense*.

#### Additional material examined.

**COSTA Rica. Alajuela**: Cantón de Grecia, Cordillera Central, Los Ángeles, camino de Los Ángeles a la Laguna de Hule, 10°17'55"N, 084°12'20"W, 740–900 m, 28 Oct 1995 (fl bud), *J. González & G. Perera 995* (CR-2 sheets, MO, NY-digital image); Cantón de San Ramón, Reserva Biológica Monteverde, río Peñas Blancas, parcela de los Enanos, 10°18'00"N, 084°43'48"W, 850 m, 02 Sep 1988 (fr), *E. Bello 332* (CR-2 sheets, F, MO, NY-digital image, USJ); Reserva Biológica Monteverde, río Peñas Blancas, 10°19'N, 084°43'W, 850 m, 06 Sep 1988 (fr), *E. Bello 353* (CR, MO, NY-digital image); Reserva Biológica Monteverde, río Peñas Blancas, 10°19'N, 084°43'W, 820 m, 10 Oct 1988 (fr), *E. Bello & E. Cruz 458* (CR, MO, NY-digital image); Reserva Biológica Monteverde, río Peñas Blancas, parcela de Badilla, 10°19'N, 084°43'W, 850 m, 22 Oct 1988 (fr), *E. Bello 473* (F, MO, NY-digital image, USJ-2 sheets); Reserva Biológica Monteverde, río Peñas Blancas, 10°18'36"N, 084°43'12"W, 900 m, 21 Apr 1993 (♂ fl), *E. Bello 5014* (CR-2 sheets); San Pedro de San Ramón, 1000 m, 06 May 1923 (♂ fl), *A.M. Brenes 19953* [Hb. Brenes 3883], (CR, F, NY-digital image); Colinas de San Pedro de San Ramón, 1050–1075 m, 27 May 1925 (fl bud), *A.M. Brenes 19955* [Hb. Brenes 4222], (CR, F, NY-digital image); Colinas de San Pedro de San Ramón, 04 Jul 1925, 1075 m, *A.M. Brenes 4827* [612], (F); Colinas de San Pedro de San Ramón, 19 May 1927 (♂ fl), *A.M. Brenes 19954* [Hb. Brenes 5445], (CR, NY-digital image); Bajos del Jamaical, Reserva de San Ramón, 700–1000 m, 10 May 1985 (♂ fl), *I. Chacón 1800* (CR-4 sheets); Reserva Forestal de San Ramón, Colonia Palmareña, 800–950 m, 19–22 Sep 1985 (fr), *J. Gómez-Laurito 10528* (CR, USJ); Reserva Forestal de San Ramón, sendero a la fila al S. O. de la Estación, 10°13'N, 084°37'W, 05 Sep 1992 (fr), *J. Gómez-Laurito 12278* (CR, F, USJ); Barranquilla, Falda Noroeste del Cerro Jabonal, 10°09'40"N, 084°39'30"W, 1500 m, 04 Nov 1997 (fr), *J. González et al. 2081* (CR-2 sheets, MO); Monteverde Reserve, Peñas Blancas river valley, Eladio Cruz farm, 10°20'N, 084°43'W, 800 m, 01 Nov 1986 (fr), *W. Haber & E. Bello 6176* (CR, NY-digital image); Reserva Biológica Monteverde, río Peñas Blancas, 10°20'N, 084°43'W, 850 m, 13 Mar 1987 (♂ fl), *W. Haber & E. Bello 6801* (CR, MO, NY-digital image); Reserva Biológica Monteverde, río Peñas Blancas, 10°20'N, 084°43'W, 800 m, 14 Apr 1987 (♂ fl), *W. Haber & E. Cruz 6979* (CR, MO, NY-digital image); Reserva Biológica Monteverde, río Peñas Blancas, 10°18'N, 084°45'W, 900 m, 21 May 1987 (fl with galls), *W. Haber & E. Bello 7169* (MO, NY-digital image); Reserva Biológica Monteverde, río Peñas Blancas, 10°20'N, 084°43'W, 820 m, 10 Jun 1997 (fr), *W. Haber & E. Cruz 7248* (CR, MO); Reserva Biológica Monteverde, río Peñas Blancas, Finca Wilson Salazar, 10°18'N, 084°43'W, 800–900 m, 20 Aug 1987 (fr), *W. Haber & E. Cruz 7391* (CR, MO, NY-digital image); Reserva Biológica Monteverde, río Peñas Blancas, Finca Wilson Salazar, 10°18'N, 084°43'W, 860 m, 20 Oct 1987 (fr), *W. Haber & E. Cruz 7508* (CR-2 sheets), *7509* (MO); Reserva Biológica Monteverde, río Peñas Blancas, Finca Wilson Salazar, 10°18'N, 084°43'W, 800 m, 06 Nov 1987 (fr), *W. Haber & E. Cruz 7691* (CR, MO, NY-digital image); Reserva Biológica Monteverde, río Peñas Blancas, 10°18'N, 084°44'W, 900, 15 Dec 1987 (fr), *W. Haber & E. Bello 7914* (CR, MO); Reserva Biológica Monteverde, río Peñas Blancas, 10°19'N, 084°43'W, 800 m, 14 Dec 1987 (♂ fl), *W. Haber & E. Bello 7899* (CR, MO, NY-digital image); San Ramón, Bosque Eterno De Los Niños, 4 km SW of La Tigra de San Carlos, valley of río La Esperanza, finca Araya Ledezma, 10°18'N, 084°37'W, 600–800 m, 01 Jul 1992 (fr), *W. Haber et al. 11232* (CR-2 sheets, MO); Reserva Forestal de San Ramón, 10°12'53"N, 084°36'28"W, 03 May 1987 (♂ fl), *G. Herrera 617* (CR, F, MO); San Ramón, Los Ángeles, Reserva de San Ramón, 2 km al Norte de la Estación, 10°12'40"N, 084°36'20"W, 1000 m, 18 Oct 1993 (fr), *G. Herrera 6604* (MO); area of the Reserva Biológica Alberto M. Brenes, 10°13'N, 084°36'W, 1010 m, 29 Apr 2001 (st), *J. Homeier & A. Wolter 723* (USJ); area of the Reserva Biológica Alberto M. Brenes, 10°13'N, 084°36'W, 1010 m, 29 Apr 2001 (st), *J. Homeier & A. Wolter 1010* (USJ); Reserva Forestal Arenal, río Peñas Blancas, Quebrada Agua Gata, Finca Francisco, 10°20'N, 084°42'W, 1200 m, 19 Sep 1990 (fr), *N. Obando 122* (CR-2 sheets, MO, NY-digital image); Reserva Biológica Monteverde, Estación Eladio’s, 10°18'30"N, 084°43'10"W, 820 m, 02 Oct 1990 (fr), *N. Obando et al. 187* (CR-2 sheets, MO, NY-digital image); San Rafael de San Ramón, 20 Oct 1969 (fr), *S. Salas et al. 1378* (USJ); Reserva de San Ramón, 13 May 1985 (♂ fl), *L. Umaña s.n.* (USJ-026360); Cantón de Upala, Bijagua, El Pilón, Cabeceras del río Celeste, 10°49'N, 084°57'W, 700 m, 21 Apr 1988 (fr), *G. Herrera 1852* (CR, MO, NY-digital image); Volcán Tenorio, Pilón, 19 Nov 1987 (fl bud), *P. Sánchez & L.J. Poveda 1282* (CR, F); Parque Nacional Guanacaste, Sector San Ramón, Dos Ríos, sendero a Níspero y Argentina, 10°52'50"N, 085°24'30"W, 550 m, 04 Mar 1995 (fr), *R. Espinoza et al. 1298* (CR-2 sheets, MO, NY-digital image); Parque Nacional Guanacaste, Nueva Zelandia, Estación San Ramón, La Campana, Dos Ríos, río Colón, 10°52'50"N, 085°24'05"W, 550 m, 23 Mar 1994 (♂ fl), *D. García 196* (CR-2 sheets, MO, NY-digital image); Cantón de San Carlos, hacia Quebrada “Corella” San Carlos, 650 m, 23 Jun 1966 (fr), *A. Jiménez 4045* (CR, F, MO, NY-digital image); La Fortuna, Finca El Jilguero, Sendero La Lava, río Aguas Calientes 0.5 km aguas arriba, 10°26'35"N, 084°42'20"W, 700 m, 23 Nov 1992 (fr), *G. Herrera 5625* (CR-2 sheets); North side Arenal Volcano, 10°28'N, 084°42'W, 800 m, 11 Apr 1974 (fr), *R. Lent 3862* (CR, F, NY-digital image). **Guanacaste**: Cantón de La Cruz, Parque Nacional Guanacaste, Estación Pitilla, al noroeste de la estación, 11°01'48"N, 085°25'12"W, 550 m, 16 Jun 1989 (fr), *B. Hammel 17495* (CR, F, MO, NY-digital image); Parque Nacional Guanacaste, Estación Pitilla, 9 km al S de Santa Cecilia, 10°59'25"N, 085°25'40"W, 700–1000 m, 06 Mar 1991 (fr), *C.O. Moraga 315* (CR-2 sheets); Parque Nacional Guanacaste, Estación Pitilla, Sendero El Mismo, Finca La Pasmompa, 11°02'00"N, 085°24'30"W, 700 m, 09 Dec 1990 (♂ fl), *P. Ríos 216* (CR, MO); Parque Nacional Guanacaste, Estación Pitilla, Fila Orosilito y Sendero El Mismo, 10°59'26"N, 085°25'40"W, 700 m, 02 Mar 1991 (fr), *P. Ríos 310* (CR-2 sheets, MO, NY-digital image); Parque Nacional Guanacaste, Estación Pitilla, Sendero El Mismo, 10°59'26"N, 085°25'40"W, 700 m, 15 Jun 1991 (♂ fl), *P. Ríos 364* (CR-2 sheets, MO); Cantón de Bagaces, Parque Nacional Rincón de la Vieja, Sendero de la toma de agua, a 3 km de la estación, 10°46'05"N, 085°17'40"W, 1000 m, 17 Sep 1990 (fr), *G. Rivera 546* (CR-2 sheets, MO, NY-2 sheets, digital image); Parque Nacional Rincón de la Vieja, Colonia Blanca, 10°48'20"N, 085°17'50"W, 1300–1600 m, 08 Nov 1990 (fr), *G. Rivera 847* (CR-2 sheets); Parque Nacional Rincón de la Vieja, Sector Santa María, Sendero La Plantación, cabeceras Quebrada Zopilote, 10°46'48"N, 085°17'24"W, 950–1100 m, 14 Aug 1996 (fr), *J. F. Morales 5667* (CR-2 sheets); Guatuso, Lago Coter, 5 km norte, Hotel Ecolodge, 10°35'20"N, 084°55'50"W, 700 m, 28 Apr 1997 (♂ fl), *G. Rivera 3005* (CR). **San José**: Reserva Forestal Los Santos, quebrada Bomba, cruce a Fila Mona y La Bomba, 09°30'00"N, 083°56'45"W, 500 m, 28 Feb 2005 (fl bud), *D. Santamaría & J.F. Morales 751* (CR); Reserva Forestal Los Santos, Dota, Fila Vega, Sendero a Fila Seca, 09°29'40"N, 083°57'30"W, 800–950 m, 03 Mar 2005 (fl bud), *D. Santamaría & J.F. Morales 900* (CR); Cantón de Pérez Zeledón, vicinity of El General, [09°23'42"N, 083°38'26"W], 1040 m, Feb 1936 (♀ fl), *A.F. Skutch 2620* (A, GH, MO, NY-digital image); Pérez Zeledón, vicinity of El General, [09°20'48"N, 083°39'27"W], 640 m, Mar 1939 (♂ fl), *A.F. Skutch 4244* (A, MO, NY-digital image); Pérez Zeledón, vicinity of El General, [09°22'20"N, 083°39'12"W], 675–900 m, Mar 1940 (♂ fl), *A.F. Skutch 4849* (A, CR, F-2 sheets, MO); basin of General, 675–900 m, 10 Feb 1942 (fl), *A.F. Skutch 5024* (F).

In view of the long history of confusion involving *Protium brenesii* and *Protium costaricense*, the following information is provided to clarify some important parameters of the latter species, as it is here interpreted:

### Protium costaricense

Taxon classificationPlantaeSapindalesBurseraceae

(Rose) Engl., Nat. Pflanzenfam., ed. 2 [Engler & Prantl] 19a: 414. 1931.

Figs 8B , 9

Icica costaricensis Rose, N. Amer. Fl. 25(3): 259. 1911. Type. COSTA RICA. [Alajuela:] Santa Clara: Las Delicias, [500 m], Jan 1897 [♀ fl], *P. Biolley 10665* [*T. Biolley* (sic), in the protologue] (holotype: US-digital image! [herbarium of Capt. John Donnell Smith, in the protologue]; isotypes: CR!, F!).

#### Habitat and distribution.

*Protium costaricense* it is known from wet forests on the Caribbean slopes of Nicaragua (Atlántico Sur and Río San Juan Departments), Panama (Colón and Panamá Provinces), and Costa Rica. In Costa Rica, it is known from throughout the Caribbean coastal plain in Alajuela and Limón Provinces. It grows in primary forest and along river or forest edges, from 0 to 200 m in elevation (reportedly up to 500 m, according to the label of the type).

#### Phenology.

Collected with staminate flowers in February and June; pistillate flowers in February, July, and August; and fruits in January and from June to December.

#### Common name.

In Nicaragua, this species is known as alcanfor (*A. Laguna 73*; *R. Rueda et al. 5338*). In Costa Rica and Panama, it goes by copal, chutra, kerosín and alcanfor (Condit et al. 2011).

#### Additional material examined.

**NICARAGUA. Atlántico Sur** [Zelaya]: Santa Fe, unión del Caño Agua Fría y Quebrada La Capilla, [11°41'N, 084°28'W], [100–200 m], 02 Oct 1982 (fr), *A. Laguna 73* (MO). **Río San Juan**: Sábalo, 1 km al norte de río San Juan, 11°02'N, 084°27'W, [100 m], 09–10 Jul 1985 (fr), *P.P. Moreno 26057* (MO); sobre el río Indio, entre San Juan del Norte Nuevo y La Casa de Narciso Orozco, incluyendo Caño Negro, 10°58'N, 083°44'W, 0–100 m, 01 Jul 1994 (♀ fl), *R. Rueda et al. 1612* (MO); Reserva Indio-Maíz, Mpio. de San Juan del Norte, Laguna de Silico, 10°51'N, 083°46'W, 0–10 m, 02 Aug 1996 (fr), *R. Rueda et al. 4850* (MO); Reserva Indio-Maíz, Mpio. de El Castillo, a lo largo del río Bartola entre el caño la Largarta y la cabecera del río Bartola, 11°16'N, 084°16'W, [50–100 m], 29 Dec 1996 (fr), *R. Rueda et al. 5127* (MO); Reserva Indio-Maíz, Mpio. de El Castillo, a 8 km de la cabecera del río Bartola, en dirección al Cerro el Diablo, 11°01'N, 084°14'W, 120 m, 04 Jan 1997 (fr), *R. Rueda et al. 5338* (MO); Reserva Indio-Maíz, Mpio. de El Castillo, 3 km al norte de la desembocadura del Caño Chontaleño, 11°05'N, 084°15'W, 24 Feb 1997 (♂ fl), *R. Rueda et al. 6285* (MO); Mpio. El Castillo, Reserva Indio-Maíz, río San Juan, entre la desembocadura del río Bartola y el Caño Sarnoso, 10°56'N, 084°20'W, 100 m, 04 Dec 1998 (fr), *R. Rueda et al. 9462* (MO). **COSTA RICA**. **Alajuela**: along road between Cañas (Guanacaste) and Upala, near río Zapote, 1.8–2.7 km south of río Canalete, ca. 100 m, 25 Jun 1976 (♂ fl), *T.B. Croat 36355* (MO, NY-digital image); San Carlos, San Luis de Cutris, 23 Sep 1983 (fr), *L.J. Poveda 3663* (USJ). **Limón**: cantón de Pococí, Refugio Nacional de Fauna Silvestre Barra del Colorado, Sardinas, 10°38'24"N, 083°43'48"W, 15 m, 25 Nov 1992 (fr), *F. Araya 61* (CR, MO, NY-digital image); Refugio Nacional de Fauna Silvestre Barra del Colorado, Puerto Lindo, 10°41'24"N, 083°39'00"W, 200 m, 24 Jul 1995 (fr), *F. Araya & J. Corrales 803* (MO, NY-digital image); Southwestern-most ridge of Cerro Coronel, NW-facing slope, just S of the río Colorado, 10°40'30"N, 083°39'30"W, 10–80 m, 17–18 Sep 1986 (fr), *G. Davidse & G. Herrera 31469* (MO); Hacienda Tapezco-Hds, La Suerte, 29 air km W of Tortuguero, 10°30'N, 083°47'W, 40 m, 20 Aug 1979 (♀ fl), *C. Davidson & J. Donahue 8514* (MO); Parque Nacional Tortuguero, Estación Agua Fría, 3 km al Sur, Sendero Real, 10°27'N, 083°34'W, 40 m, 18 Jan 1988 (fr), *R. Robles 1624* (MO, NY-digital image); Refugio Nacional de Fauna Silvestre Barra del Colorado, sector Cocorí, 30 km N de Cariari, 10°35'40"N, 083°48'00"W, 100 m, 15 Jun 1991 (fr), *E. Rojas 227* (CR, MO, NY-digital image); río Santa Clara, 1 ½ mi NW. of Los Diamantes, ne. of Guápiles, ca. 980 ft [298 m], 18 Aug 1961 (fr), *G.B. Rossbach 3823* (GH); Cerro Coronel, E of río Zapote, 1 km of río Colorado, 10°40'N, 083°40'W, 10–40 m, 13–14 Sep 1986 (fr), *W.D. Stevens & O.M. Montiel 24333* (MO); Pueblo Nuevo, 17 km NE of Guácimo, 10°20'N, 083°36'W, 100 m, 07 Sep 1994 (fr), *K. Thomsen 1112* (CR, NY-digital image). **PANAMA. Colón**: Donoso, río Hoja, UTM: E544656; N985023 [08°54'39"N, 080°35'38"W], 23 Aug 2009 (fr), *B. Araúz & J. De Gracia 2118* (MO). **Panamá**: Zona del Canal: Barro Colorado Island, Frank Drayton Trail, [09°09'15"N, 079°51'05"W], [10–150 m], 22 May 1968 (fl bud), *T.B. Croat 5769* (MO); Barro Colorado Island, William Morton Wheeler Trail, [09°09'20"N, 079°51'10"W], [10–170 m], 22 Sep 1968 (fr), *T.B. Croat 6295* (MO); Barro Colorado Island, Drayton House, [09°08'29"N, 079°50'35"W], [10 m], 28 Feb 1969 (st), *T.B. Croat 8262* (MO); Barro Colorado Island, Drayton House, [09°08'29"N, 079°50'35"W], [10 m], 16 Jul 1970 (fr), *T.B. Croat 11337* (F, MO); Drayton House, [09°08'29"N, 079°50'35"W], [0–5 m], 28 Aug 1970 (fr), *T.B. Croat 11939* (MO); Barro Colorado Island, James Zetek Trail, [09°09'31"N, 079°52'05"W], [10–100 m], 07 Jun 1971 (♂ fl), *T.B. Croat 14926* (F, GH, MO-2 sheets); Barro Colorado Island, Drayton House, 06 Jul 1969 (fr), *R. Foster 1093* (F, GH); Pipeline Road, 4 mi. N of Gamboa, [09°10'N, 079°46'W], [50–100 m], 21 Dec 1971 (st), *A.H. Gentry 3230* (MO); Pipeline Road, [09°10'N, 079°46'W], 100 m, 10 Aug 1971 (fr), *E. Lao et al. 16* (GH, MO-2 sheets); Pipeline Road, 16.6 km from beginning of road, N along río Agua Salud, [09°14'38"N, 079°48'57"W], 0–100 m, 22 Sep 1974 (fr), *S.A. Mori & J.A. Kallunki 2039* (GH, MO); along río Mendosa near Pipeline Road, 8 km NW of Gamboa, [09°09'36"N, 079°44'44"W], 95 m, 01 Nov 1973 (fr), *M. Nee 7743* (MO); Isla Barro Colorado, 120 m, 12 Oct 2006 (fr), *R. Pérez & S. Aguilar 1632* (MO); Barro Colorado Island, W of Drayton House, [09°08'29"N, 079°50'35"W], [0–10 m], 16 Feb 1932 (♀ fl), *R.H. Woodworth & P.A. Vestal 605* (A, MO).

The pseudarils of *Protium costaricense* are reported to have a pleasant flavor (*L.J. Poveda 3663*, USJ).

## Supplementary Material

XML Treatment for Protium aguilarii

XML Treatment for Protium hammelii

XML Treatment for Protium brenesii

XML Treatment for Protium costaricense
